# An Ecological Analysis of the Herbivory-Elicited JA Burst and Its Metabolism: Plant Memory Processes and Predictions of the Moving Target Model

**DOI:** 10.1371/journal.pone.0004697

**Published:** 2009-03-11

**Authors:** William Stork, Celia Diezel, Rayko Halitschke, Ivan Gális, Ian T. Baldwin

**Affiliations:** Department of Molecular Ecology, Max Planck Institute for Chemical Ecology, Beutenberg Campus, Jena, Germany; Gothenburg University, Sweden

## Abstract

**Background:**

Rapid herbivore-induced jasmonic acid (JA) accumulation is known to mediate many induced defense responses in vascular plants, but little is known about how JA bursts are metabolized and modified in response to repeated elicitations, are propagated throughout elicited leaves, or how they directly influence herbivores.

**Methodology/Principal Findings:**

We found the JA burst in a native population of *Nicotiana attenuata* to be highly robust despite environmental variation and we examined the JA bursts produced by repeated elicitations with *Manduca sexta* oral secretions (OS) at whole- and within-leaf spatial scales. Surprisingly, a 2^nd^ OS-elicitation suppressed an expected JA burst at both spatial scales, but subsequent elicitations caused more rapid JA accumulation in elicited tissue. The baseline of induced JA/JA-Ile increased with number of elicitations in discrete intervals. Large veins constrained the spatial spread of JA bursts, leading to heterogeneity within elicited leaves. 1^st^-instar *M. sexta* larvae were repelled by elicitations and changed feeding sites. JA conjugated with isoleucine (JA-Ile) translates elicitations into defense production (e.g., TPIs), but conjugation efficiency varied among sectors and depended on *Na*WRKY3/6 transcription factors. Elicited TPI activity correlated strongly with the heterogeneity of JA/JA-Ile accumulations after a single elicitation, but not repeated elicitations.

**Conclusions/Significance:**

Ecologically informed scaling of leaf elicitation reveals the contribution of repeated herbivory events to the formation of plant memory of herbivory and the causes and importance of heterogeneity in induced defense responses. Leaf vasculature, in addition to transmitting long-distance damage cues, creates heterogeneity in JA bursts within attacked leaves that may be difficult for an attacking herbivore to predict. Such unpredictability is a central tenet of the Moving Target Model of defense, which posits that variability in itself is defensive.

## Introduction

Herbivory by leaf-chewers elicits a rapid, but transient, accumulation of the oxylipin, jasmonic acid (JA), in the attacked leaves of vascular plants. JA and its metabolites elicit the expression of defense-related genes and large-scale changes in the transcriptome, proteome, and metabolome after leaf wounding and herbivory [1]–[3] and is associated with a repression of growth and activation of defense-related processes [4]–[7]. Regulatory roles have also been suggested for members of the extensive jasmonate family [8]–[10], including a central role for an isoleucine conjugate (JA-Ile) that promotes the SCF^COI1^-mediated degradation of JAZ repressors of transcription factors that regulate JA-responsive genes in *Arabidopsis thaliana* [11], [12]. In addition to regulatory roles in herbivore-attacked tissues, jasmonates have an unidentified but essential role in the elicitation of the systemic defense response [13].

Mechanical simulations of herbivory have been effectively employed to elucidate the complicated temporal dynamics of the various oxylipins that account for the responses elicited by the JA signaling network. Such simulations often consist of single, whole-leaf elicitations, followed by homogenization of the elicited leaf tissues for analysis. These techniques are suited for identifying key regulatory nodes in the JA signaling network and studying whole-plant systemic defense, but do not capture the temporal patterns and spatial heterogeneity of the responses elicited by a feeding herbivore. Some recent studies have demonstrated the effect of repeated elicitations on plant defense. A mechanical wounding robot programmed to simulate the timing of continuous Lepidopteron herbivory was able to replicate the induced volatile profile elicited when *Spodoptera littoralis* larvae attack lima bean plants [14], [15]. Other studies have highlighted important spatial heterogeneity in the induced responses. In *A. thaliana*, more polar jasmonate metabolites were found in vascular tissues of induced leaves [16]. MAPK signaling in *Nicotiana attenuata* after elicitation of different leaf quarters was activated heterogeneously within elicited leaves [17]. In tomato, direct vascular connectivity to a crushed leaf determined the induction of TPIs in systemic leaves and contributed to spatial heterogeneity of induced TPIs within those systemic leaves [18]. The factors responsible for heterogeneity within elicited leaves, however, remain largely unexplored. Recently, the non-uniform distribution of glucosinolates within *A. thaliana* leaves was shown to influence the feeding behavior of Lepidopteran herbivores [19]. Characterizing the temporal patterns and spatial heterogeneity of induced defenses at an appropriate scale is therefore critical for understanding plant-herbivore interactions.

*N. attenuata* provides an ecologically informed model for reconsidering temporal patterns and spatial heterogeneity of the JA-mediated induced defense response, particularly in response to Lepidopteran herbivory. Annually established populations of this native tobacco encounter highly variable herbivore communities as plants germinate from seed banks that respond to smoke cues from wildfires in the Great Basin Desert [20], [21]. *N. attenuata* is known to respond specifically to attack from the larvae of the specialist Lepidopteran herbivore, *Manduca sexta* [22]. Wound-induced JA accumulation is amplified by *M. sexta* oral secretions (OS), but JA-dependent nicotine synthesis is inhibited by ethylene when this nicotine tolerant herbivore initiates feeding [23]. JA-Ile dependent TPI activity effectively reduces larval growth rates [24], [25], but expression of TPIs is costly for *N. attenuata* in the absence of herbivores [26].

Elicited JA accumulation in *N. attenuata* is extremely dynamic: the initial JA burst attains maximum values 45–60 min after OS elicitation and is quickly metabolized, with JA levels returning to about 1/8^th^ of peak levels within 2 h and then slowly returning to undetectable levels [27]. In comparison, *A. thaliana* maintains highly induced levels of JA for at least 4 h after wounding [28], although a biphasic accumulation pattern has been reported with induced levels increasing even days after a single wounding [29]. Unlike in *N. attenuata*, wound-induced JA accumulation in *A. thaliana* is not known to respond to herbivore-specific cues. The rapid waxing and waning of the JA burst in *N. attenuata* may be of particular importance in tailoring defense responses in response to sustained herbivore attack.

Here, we characterize and test several aspects of *N. attenuata*'s JA burst and its role in plant defense. We focus on responses in the laminal tissues subtended by leaf vasculature because these are the tissues primarily attacked by early-instar *M. sexta* larvae on *N. attenuata*. We report that the JA burst in a native population of genetically heterogeneous plants, exposed to natural abiotic stresses thought to influence JA signaling (e.g., UV-B), is surprisingly robust. We demonstrate that repeated elicitations alter the characteristic patterns of induced JA accumulation at both whole- and within-leaf scales. Using a scaled-down elicitation method that mimics herbivory by 1^st^ instar *M. sexta* larvae, we show that large vascular structures (the midrib and secondary veins) inhibit the spread of JA bursts and JA metabolites within elicited leaves, but that connections between laminal leaf sectors via secondary veins could account for the apparent unpredictability of the between-sector spread of JA bursts. We also show that larvae are repelled from OS-elicited leaf sectors in a timeframe correlating to the initial JA burst itself, rather than changes in the plant defense profile induced by the JA burst; larvae changed feeding sites from elicited to un-elicited leaf sectors well before the JA bursts are translated into the expression of JA-associated defenses such as trypsin proteinase inhibitors (TPIs). JA is conjugated with Ile to form JA-Ile, which translates elicitations into defense production (e. g. TPIs) [30]. The efficiency of conjugation of JA with Ile varied among sectors, with the laminal sector at the base of the leaf having the lowest efficiency. To explore the role of transcription factors in the conjugation efficiency, we used recently characterized, transformed *N. attenuata* plants silenced in the expression of two WRKY transcription factors (*Na*WRKY3 and 6) that mediate the translation of OS elicitations into oxylipin signaling [31].

The results of this work highlight the importance of studying the elicitation of defense responses at spatial scales relevant to the attacking herbivore and suggest that the JA burst can itself function as a defense. We consider the temporal and spatial dynamics of the responses in a framework of memory formation. The analysis also demonstrates that leaf vasculature, in addition to transmitting systemic signals among attacked leaves, creates heterogeneity of the JA burst within attacked leaves, which may be difficult for the attacking herbivore to predict. Unpredictability of defense responses is a central tenet of the Moving Target Model of induced plant defense [32].

## Methods

### Characterizing the N. attenuata JA burst in a native population

Since oxylipin signaling is thought to be influenced by abiotic stresses such as drought, wind stress and UV-B exposure [33], [34], we examined the robustness of the OS-elicited JA burst to environmental perturbation by eliciting the 1^st^ or 2^nd^ fully expanded leaf from each of 65 rosette-stage plants growing in a native population in the Great Basin Desert at Lytle Ranch Preserve, St. George, Utah, USA. Elicited plants were likely highly genetically heterogeneous, as was determined by an AFLP analysis of native *N. attenuata* plants growing in the same area the previous year [35]. Elicitation occurred just before elongation of rosette-stage plants and consisted of making 3 rows of puncture wounds with a pattern wheel on each side of the midrib (4 puncture wounds per cm of leaf lamina as in [36]) and immediately applying either 10 µl of *M. sexta* OS diluted 1∶5 in distilled H_2_O or 10 µl distilled H_2_O. Whole leaves of replicate groups of 5 plants each were then harvested at 30 min increments after elicitation and immediately stored and shipped on dry ice for JA quantification. To visualize the elicited JA burst in this native population, we constructed a 4 hr kinetic of JA accumulation after elicitation with OS or the water control by plotting the mean value of quantified JA in samples from each harvested replicate group along a horizontal axis based on time elapsed before harvest after the initial elicitation.

### Repeated simulations of M. sexta herbivory at the whole-leaf scale

Arthropod herbivores—once successfully established on a plant—rarely stop feeding after the initial attack. We examined the effect of repeated herbivory events on the highly reproducible JA burst characterized in this and other studies. Repeated herbivory events could affect JA accumulations in several ways: (1) JA levels might be unresponsive or repressed after repeated herbivory events, (2) repeated herbivory events could maintain a stable induced level of JA, or (3) lead to increasing JA levels.

Glasshouse plants were germinated from the 17^th^ inbred generation of seeds originally collected from a native population in Utah collected at the DI ranch in 1988 [20]. Seeds were germinated on Gamborg B5 media under sterile conditions, with gibberellic acid and 1∶50 diluted liquid smoke (House of Herbs, Passaic, NJ, USA) as described in [37]. 10-d-old seedlings were planted individually in soil in Teku pots. 10 d later, early rosette stage plants were transferred to soil in 1 L pots and grown in a glasshouse at 26 to 28°C under 16 h of light per day (Philips Sun-T Agro 400 W sodium lights, www.nam.lighting.philips.com). Rosette-stage plants were elicited before elongation. A single elicitation consisted of 1 row of pattern wheel wounds on each side of the midrib, which were immediately treated with 10 µl of 1∶5 diluted OS per row; rows of successive elicitations were added on the leaf-margin side of the previous row. Whole leaf replicate groups were harvested at 30 min increments after the last elicitation and immediately frozen in liquid N_2_ and stored at −80°C until JA quantification.

To determine how JA accumulation after the initial burst is modified by subsequent herbivory events, we OS-elicited the 1^st^ or 2^nd^ fully expanded leaf of glasshouse-grown *N. attenuata* once per h and constructed 6 h kinetics of JA accumulation in response to 1, 2, 3, and 4 elicitations using a similar method as above: mean quantified JA level in samples from each harvested replicate group were plotted along an axis based on time elapsed before harvest after the last elicitation.

### Simulation and analysis of early-instar herbivory

*M. sexta* larvae consume 98% of their total food intake during the 5^th^ instar [38] and can easily consume whole plants; therefore, induced plant defenses must be targeted at early, more vulnerable instar stages in order to be effective against these voracious herbivores. Therefore, we were interested in characterizing the OS-elicited JA burst on a spatial scale relevant to the feeding behavior of early-instar larvae. Observations of feeding, 1^st^ instar *M. sexta* larvae suggested that the spatial scale of attack could be simulated by piercing the leaf lamina with a sharp needle and immediately adding 1 µl of undiluted OS. This localized elicitation method allowed us to test two aspects of JA accumulation. First, we determined whether the modifications of JA accumulation by repeated elicitations that we had observed at the whole-leaf scale also occurred on a smaller within-leaf spatial scale. We tested the observed JA accumulation pattern against an additive model to identify specific alterations of expected JA accumulation by multiple elicitations. We used the JA kinetic generated from a single, localized elicitation to create a predictive model for JA accumulation in response to repeated elicitations. Based on the hypothesis that successive elicitations do not alter biosynthetic and metabolic fates of JA, this model predicted net JA levels at time *t* after *n* elicitations by summing the JA levels resulting from a series of theoretical single elicitations.

**Figure 1:**
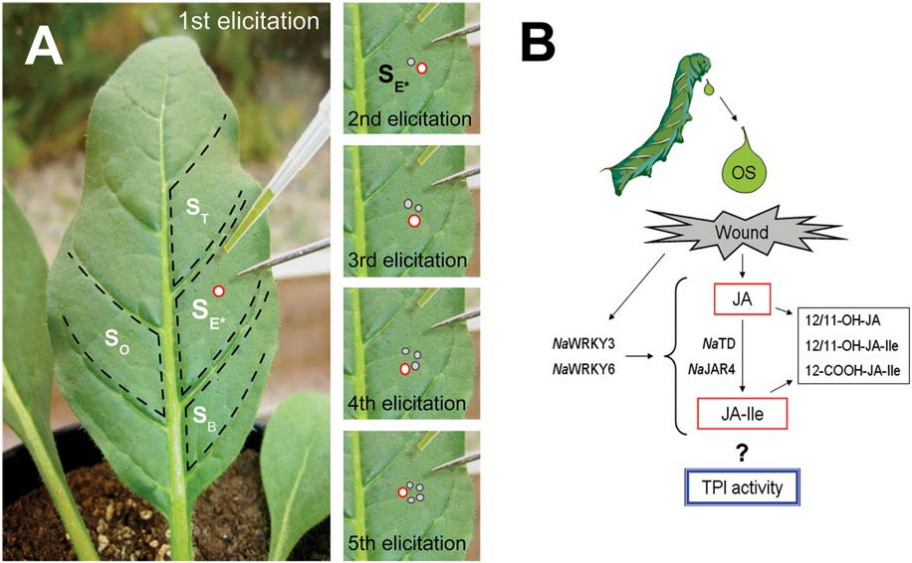
Repeated elicitations of *N. attenuata* leaves at the scale of a feeding, early-instar *M. sexta*. (A) 1 µl of undiluted *M. sexta* OS was applied to a needlepoint wound in a single middle sector of the leaf lamina. This elicitation was repeated 5 times (1 per h). Four laminal sectors were dissected and extracted (S_E*_, S_T_, S_B_, and S_O_) for jasmonate (JA) and TPI quantification. (B) OS elicitation results in rapid accumulation of JA, some of which is rapidly conjugated to Ile. Conjugation requires both *Na*TD and *Na*JAR4; silencing *Na*JAR4 and *Na*TD transcript accumulation decreases TPI activity [25], [30]. Bioactive JA and JA-Ile can be hydroxylated at C-12 or C-11, or carboxylated at C-12. *Na*WRKY3 and *Na*WRKY6 mediate the accumulation and metabolism of JA by influencing processes upstream of JA biosynthesis [31].

Second, we measured the accumulation of jasmonates within different sectors of an elicited leaf. Wu et al. showed that the patterns of OS- induced JA accumulations differed among leaf quarters cut along and across the midrib, depending on the leaf quarter that was OS-elicited [17]. We hypothesized that the midrib and the large secondary veins that run roughly perpendicular to the lengthwise gradient constrain the spatial spread of the OS-elicited JA burst across the leaf lamina, leading to heterogeneity of JA accumulation within an elicited leaf. To test this hypothesis, we dissected elicited leaves into intra-vein laminal sectors defined by the midrib and secondary veins (Fig. 1) and quantified jasmonate accumulations in each sector individually.

Localized elicitations were created by pushing a needlepoint through the 2^nd^ full laminal sector (from the base of the leaf) of rosette-stage, glasshouse-grown *N. attenuata*, and adding 1 µl of undiluted OS (Fig. 1A). 2 non-orthostichous leaves per plant were elicited for jasmonate quantification kinetics [39]; a sub-set of samples were paired so as to compare potential whole-plant systemic effects in jasmonate accumulation. We repeated this elicitation 5 times (1 per h) within the focal sector; successive elicitations proceeded in a clockwise circle within the elicited laminal sector. Replicate groups were harvested in 30 min increments from the time of the last elicitation. During harvest, leaves were dissected into sectors divided by the midrib and secondary vasculature and 4 sectors were saved for jasmonate quantification over a 6 h period: the elicited laminal sector (referred to as S_E*_), the sector adjacent to S_E*_ towards the base of the leaf (**S_B_**), the sector adjacent to S_E*_ towards the tip of the leaf (**S_T_**), and the sector opposite from S_E*_ across the leaf midrib (**S_O_**). Plants were grown under identical conditions as those used for the whole-leaf glasshouse elicitation, but came from the 30^th^ inbred generation of seeds originally collected at Lytle Ranch Preserve. All laminal sectors were immediately (<1 min after harvest) frozen in liquid N_2_ and stored at −80°C until quantification of JA and derivatives. This analysis entailed the quantification of 4 sectors each from 112 individual leaf samples.

### Identifying molecular mechanisms regulating the JA burst and its metabolism

Transcription factors *Na*WRKY3 and *Na*WRKY6 are involved in maintaining induced JA levels at the whole-leaf scale during continuous herbivory [31]. We used stably transformed lines, which were silenced in the expression of either *Na*WRKY6 or both *Na*WRKY3 and *Na*WRKY6 by RNAi as described in [31] to determine whether these transcription factors also are responsible for mediating the temporal patterns of JA accumulation that we observed at the within-leaf spatial scale. Seeds from these stably transformed lines were germinated and grown as described for glasshouse wild-type plants, and the within-laminal sector elicitation scheme and harvest was used to construct JA and derivative accumulation kinetics. Control wild-type plants were germinated and grown alongside transformed plants.

### Quantification of JA and its derivatives

For field- and glasshouse-grown plants elicited at the whole-leaf scale, we were interested in testing the effect of certain factors (environmental perturbation, repeated elicitations) on the JA burst. However, for the localized elicitation scheme, we were additionally interested in how the JA burst is metabolized in order to determine the contribution of metabolism to the observed temporal and spatial heterogeneity.

Since the JA burst rapidly wanes after a single elicitation, we quantified 5 additional metabolites of JA, some of which are thought to be the elicitors of defense responses (Fig 1B). As JA accumulates in response to simulated or real herbivory, *Na*JAR4 catalyzes its conjugation to isoleucine (Ile) to form JA-Ile; silencing this enzyme impairs the activation of TPI defenses, as does silencing threonine deaminase (*Na*TD) which supplies the Ile used in the conjugation [25], [30]. JA is thought to be inactivated by hydroxylation at carbon 12 or 11 (combined relative quantities are reported here as 12/11-OH-JA) [40], [41]. Recently, a similarly hydroxylated JA-Ile (combined relative quantities are reported here as 12/11-OH-JA-Ile) and a dicarboxylic jasmonate (12-COOH-JA-Ile) have been reported *in planta* [16], [42]. Quantifying this group of jasmonates allowed us to observe accumulation of the important signaling molecules JA and JA-Ile, as well as their metabolism, to better understand the processes responsible for the waning of the JA burst.

Jasmonates from leaf tissue were extracted in 1 ml ethyl acetate spiked with D_2_-JA and ^13^C_6_-JA-Ile internal standards (ISTDs). Whole-leaf samples were first homogenized by grinding in liquid nitrogen and extracted in ∼100 mg aliquots (exact weight of each aliquot was recorded). Dissected laminal sectors were weighed before homogenization. Samples were ground to a fine powder with porcelain beads to ensure thorough homogenization (Fast Prep homogenizer, www.thermo.com), extracts were centrifuged in a microcentrifuge (*rcf* 16,100×g, 20 min, 4°C), and supernatants evaporated to dryness. Dried extracts were re-suspended in 70% methanol for HPLC-MS/MS analysis. Amounts of ISTD used for extraction were adjusted between experiments to match expected values of jasmonates in the replicates: 100 ng per sample for whole-leaf extractions, 10 ng per sample for the WT-only laminal sector extractions, and 50 ng per sample for the WT/ir-*wrky6*/ir-*wrky3/6* laminal sector extractions.

10 µl extract aliquots were analyzed by reverse-phase HPLC coupled to a Varian 1200 L triple-quad mass spectrometry (MS/MS) system (www.varianinc.com). Multiple reaction monitoring (MRM) was conducted on parent-ion/product-ion selections after negative ionization: 213/59 (D_2_-JA), 209/59 (JA), 225/59 (12/11-OH-JA), 328/136 (^13^C_6_-JA-Ile), 322/130 (JA-Ile), 338/130 (12/11-OH-JA-Ile), 352/130 (12-COOH-JA-Ile). The area beneath the MRM product ion peak was recorded for detected analytes and ISTDs. MRM for 12/11-OH-JA and 12/11-OH-JA-Ile returned 2 separate peaks reflecting the different position of OH moieties: retention time (RT) for 12-OH-JA = 5.317 min, RT_11-OH-JA_ = 5.564 min, RT_12-OH-JA-Ile_ = 5.671 min, RT_11-OH-JA-Ile_ = 5.963 min. For these analytes, the areas beneath the 2 peaks were combined. The concentration of analytes was quantified by multiplying the analyte∶ISTD ion peak area ratio by the mass of ISTD added during the extraction. D_2_-JA was used as ISTD for 12/11-OH-JA and ^13^C-JA-Ile was used as ISTD for 12/11-OH-JA-Ile and 12-COOH-JA-Ile and values relative to the respective ISTDs were reported.

### Testing caterpillar feeding behavior in response to an elicited JA burst

The OS-elicited JA burst is known to be essential for the activation of defense responses, which in turn influence the feeding patterns of *M. sexta* larvae among leaves on a plant [38], [43], [44]. To determine if the rapid accumulation of JA in response to herbivory, or later JA-associated defense processes, influenced caterpillar feeding behavior within a leaf, we placed larvae on the underside of WT *N. attenuata* leaves that had been elicited with a single, needle-point wound and 1 µl of undiluted *M. sexta* OS on S_E*_ (1) 2 h before larval placement, (2) 15 min before larval placement, or (3) not elicited.

Eggs of *M. sexta* obtained from North Carolina State University (Raleigh, NC, USA) were hatched at 24 to 26°C under 16 h light and fed for 36 h on stably transformed as-*lox3 N. attenuata* plants before the experiment started to ensure that larvae would not be exposed to JA-induced responses during their first leaf meal (inserted *lox3* anti-sense construct characterized in [43]). 36-h-old individuals were starved in a plastic box for 30 min before the experiment to promote immediate feeding. The three leaf treatments (elicitation 2 h prior to experiment, elicitation 15 min prior, and control) were paired on each plant; the 1^st^, 2^nd^, and 3^rd^ fully expanded leaves of partially elongated plants were used and treatments were moved among the different leaves on replicate plants. Caterpillars were observed every 5 min for 1 h to record location and time of the first feeding event. Feeding was constrained to S_E*_ to ensure that the first tissues larvae sampled on the experimental plants were from S_E*_. Larvae that failed to feed within 1 h were discarded from the analysis. Groups of 7 or 8 plants (3 caterpillars each) were observed each day on 4 consecutive days.

### Radial diffusion assay of trypsin proteinase inhibitor activity due to single or repeated elicitations

To understand how a leaf integrates the temporal and spatial dynamics of the JA (or JA-Ile) burst into a defense response effective against early instar *M. sexta* larvae at a molecular level, we measured trypsin proteinase inhibitor (TPI) activity at 12 and 24 h after a single, localized elicitation and 5 repeated elicitations in each of the laminal sectors described above. Wild-type *N. attenuata* were grown and elicited in the glasshouse as described above. Replicate leaves were dissected into laminal sectors and frozen in liquid N_2_ at 12 or 24 h after the time of 1^st^ elicitation.

Water-soluble proteins were extracted from ground leaf tissue [45] and total protein content in each sample was determined by the Bradford assay against serial dilutions of an immunoglobulin G standard (Sigma-Aldrich, www.sigmaaldrich.com). Approx. 25 µl of protein extract from each sample was loaded into wells on gel plates containing trypsin (Sigma-Aldrich) dissolved in plant agar. Serial dilutions of soybean TPI (Sigma-Aldrich) were used as standard on each gel plate. Active TPI from samples was allowed to radially diffuse out from each loaded well for 14 h. Plates were then stained to reveal extent of TPI diffusion [45], [46]; TPI activity was quantified by standardizing the diameter of the diffusion ring from each sample against the soybean TPI curve and dividing by total water-soluble protein [47]. Statistical analyses (ANOVA and Fisher's PLSD) were performed with StatView (Adept Scientific, citewise.adeptscience.co.uk/statview/).

## Results

### The OS-elicited JA burst is highly robust and repeated elicitations result in non-additive JA accumulations

**Figure 2:**
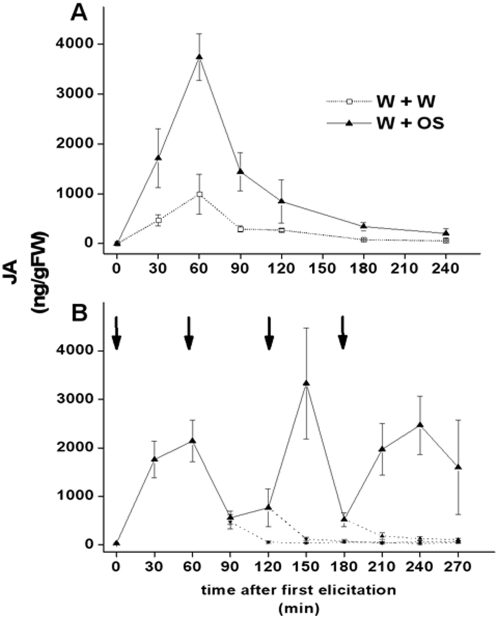
Whole-leaf elicitation: a single OS elicitation induces a robust, transient JA burst even in a genetically heterogeneous native population of *N. attenuata*, but JA accumulations in response to repeated elicitations are not additive. (A) A single whole-leaf OS elicitation of leaves on rosette stage plants from a native population growing at the Lytle Ranch Preserve, St. George, Utah, USA, resulted in a robust, transient JA burst similar to that observed in genetically homogeneous glasshouse-grown plants. Leaves were elicited by wounding with a pattern wheel and the resulting puncture wounds were immediately treated with either water (open squares, dotted line) or 10 µl of 1∶5 diluted *M. sexta* OS in water (solid triangles, solid line). (B) Repeated whole-leaf OS-elicitations (1 per h) of leaves from rosette stage, inbred plants grown in the glasshouse suggest competition between suppression and maintenance of JA accumulation. Arrows indicate time of each elicitation. All values represent mean±S.E. (n = 5).

The kinetics of the OS-elicited JA burst measured from these genetically heterogeneous native plants that had grown in an environment replete with insults known to elicit JA bursts was remarkably similar to those quantified in over 30 individual glasshouse experiments over the past 13 years (for example [48]): the JA burst attained a distinctive peak 60 min after elicitation followed by a rapid decline to baseline levels within 3 h (Fig. 2A). The mean peak JA concentration, at >3500 ng/g FM, was higher than those attained in similar glasshouse experiments.

The JA kinetic resulting from whole-leaf elicitations of glasshouse-grown plants did not exactly match any of the predicted patterns (Fig. 2B). For example, re-elicitation of leaves already at the peak of JA accumulation (1 h after 1^st^ elicitation) surprisingly decreased JA levels and did not increase or even sustain the JA peak. However, subsequent elicitations again induced JA bursts.

### Nonadditive JA accumulations in response to repeated elicitations

In response to localized elicitation, the elicited laminal sector (S_E*_) accumulated peak JA levels (mean JA in S_E*_ = 4800±90 ng/g FM) that were higher than those quantified in homogenized whole-leaf samples, which suggests that elicitation did not elicit a JA burst uniformly across the leaf lamina.

**Figure 3:**
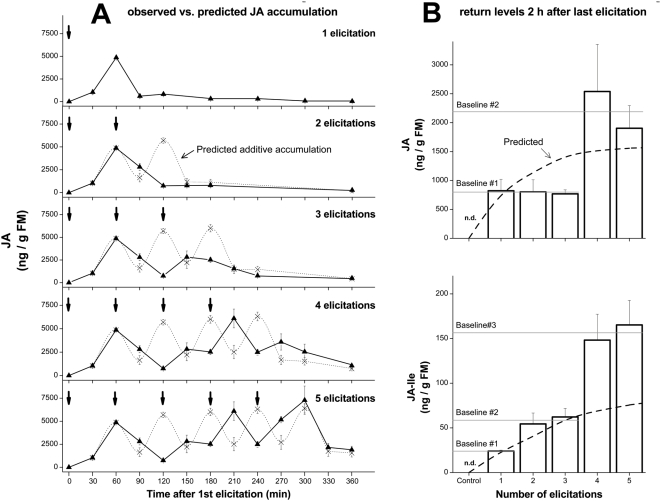
Accumulation of JA in elicited sectors after repeated elicitations is not strictly additive, but baseline JA and JA-Ile increase in discrete steps. (A) Observed patterns of OS-elicited JA accumulation (triangles, solid lines) differ from those predicted from the addition of repeated single elicitations (x's, dotted lines). Arrows indicate time of elicitation (1 per h). The JA burst was suppressed after the 2^nd^ elicitation, but returned after the 4^th^ and 5^th^ elicitations. (B) JA accumulation due to a single elicitation returned to a lower baseline level after the initial burst. The additive model in Panel A predicted that this baseline JA level would gradually increase to a saturated level as regular elicitations resulted in a regular pattern of JA bursts. However, observed baseline return levels of JA and JA-Ile 2 h after repeated elicitations (open bars) increased in discrete steps. All values represent JA or JA-Ile mean+S.E. (n = 4).

**Figure 4:**
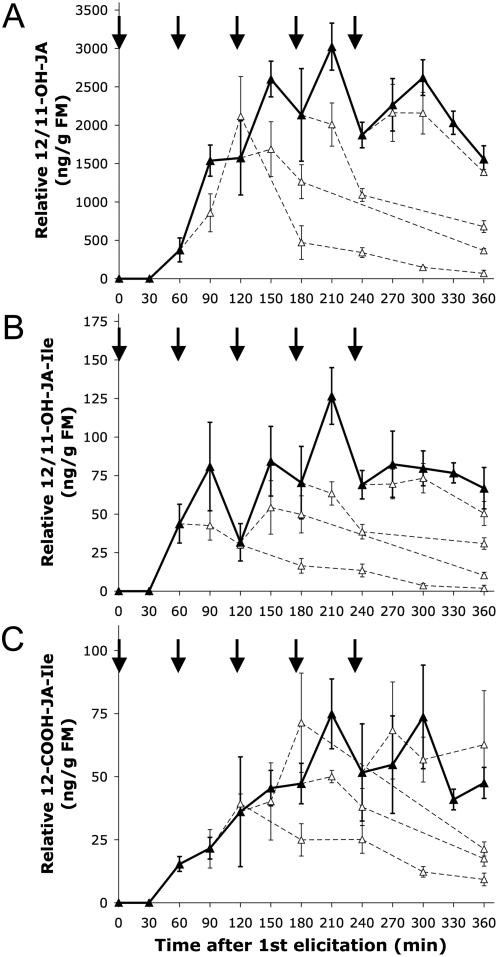
Accumulation of hydroxylated JA and JA-Ile (at C-12 or C-11) and carboxylated JA-Ile (at C-12) in elicited laminal sectors (S_E*_) after repeated elicitations. (A) Hydroxylated JA begins to accumulate about 60 min after OS-elicitation, when JA attains maximum levels. Increases in JA accumulation after repeated elicitations could result from limited or suppressed metabolism of JA. However, repeated elicitations result in immediate increases in hydroxylated JA (closed triangles, solid lines) compared to hydroxylated JA levels had the elicitations not occurred (open triangles, dashed lines), indicating that hydroxylation of JA is not limiting (at least until the 5^th^ elicitation). Arrows indicate time of elicitation (1 per h). Values represent relative 12/11-OH-JA mean±S.E. (n = 4) compared to a JA standard. (B) Relative accumulations of 12/11-OH-JA-Ile in response to 5 repeated elicitations in elicited laminal sector (S_E*_). Repeated elicitations result in immediate increases in hydroxylated JA-Ile (closed triangles, solid line) compared to hydroxylated JA-Ile levels had the elicitations not occurred (open triangles, dashed lines). Arrows indicate time of elicitation (1 per h). Values represent relative 12/11-OH-JA-Ile mean±S.E. (n = 4), compared to a JA-Ile standard. (C) Relative accumulations of 12-COOH-JA-Ile in response to repeated elicitations in elicited laminal sector (S_E*_). Repeated elicitations result in immediate increases in 12-COOH-JA-Ile (closed triangles, solid line) compared to 12-COOH-JA-Ile levels had the elicitations not occurred (open triangles, dashed lines). Arrows indicate time of elicitation (1 per h). Values represent relative 12/11-OH-JA mean±S.E. (n = 4), compared to a JA-Ile standard.

In response to repeated elicitations at a frequency of 1 per h, observed levels of JA within S_E*_ did not match the predicted additive model, implying that JA biosynthetic and metabolic rates in S_E*_ are altered by repeated elicitations (Fig. 3A). For simplicity, we only describe JA accumulation patterns; JA-Ile accumulation followed JA accumulation closely. As in the whole-leaf elicitation, a 2^nd^ elicitation in S_E*_ decreased JA accumulations and the predicted 2^nd^ JA burst was not observed. The 2^nd^ elicitation increased the accumulation of 12/11-JA-OH, so that at the 90 min harvest, mean values were almost 80% higher after 2 elicitations than after 1 (Fig. 4A). Immediate increases in 12/11-JA-OH were also apparent after the 3^rd^ and 4^th^ elicitations, but not after the 5^th^.

Differences between observed and predicted patterns of JA accumulation were clearer after the 3^rd^, 4^th^, and 5^th^ elicitations: net JA increased immediately after each successive elicitation instead of following the initial decrease predicted from the model (Fig. 3A). After the 4^th^ and 5^th^ elicitations, this rapid accumulation led to peak means that were at least as high as those predicted from the model, but JA accumulation following the 3^rd^ elicitation (as after the 2^nd^ elicitation) never reached the predicted levels and thereby resembled the reduction in net JA accumulation observed after the 2^nd^ elicitation in the whole-leaf elicitation experiments.

Relative accumulation of 12/11-OH-JA-Ile and 12-COOH-JA-Ile in S_E*_ were also detected, and net levels of both increased in response to repeated elicitations (Fig. 4B and Fig. 4C). In general, 12/11-OH-JA-Ile accumulation was responsive to each successive elicitation except for the 5^th^, but high variance made it difficult to detect more specific patterns.

### Repeated elicitations result in discrete increases in basal levels of JA and JA-Ile

After attaining peak values in response to a single elicitation, JA and JA-Ile in S_E*_ returned to a new, slightly elevated baseline level (∼15% of peak) after 2 h. Successive elicitations increased this baseline, not in a gradually saturating pattern as predicted by the additive model, but in discrete steps (Fig. 3B). The JA baseline was only raised to a higher tier after 4 elicitations and returned to that same tier after 5 elicitations. The JA-Ile baseline was elevated after the 2^nd^ elicitation and then again after the 4^th^ elicitation.

### JA and JA-Ile bursts decrease dramatically in laminal sectors separated from the elicitation site by veins and midribs

**Figure 5:**
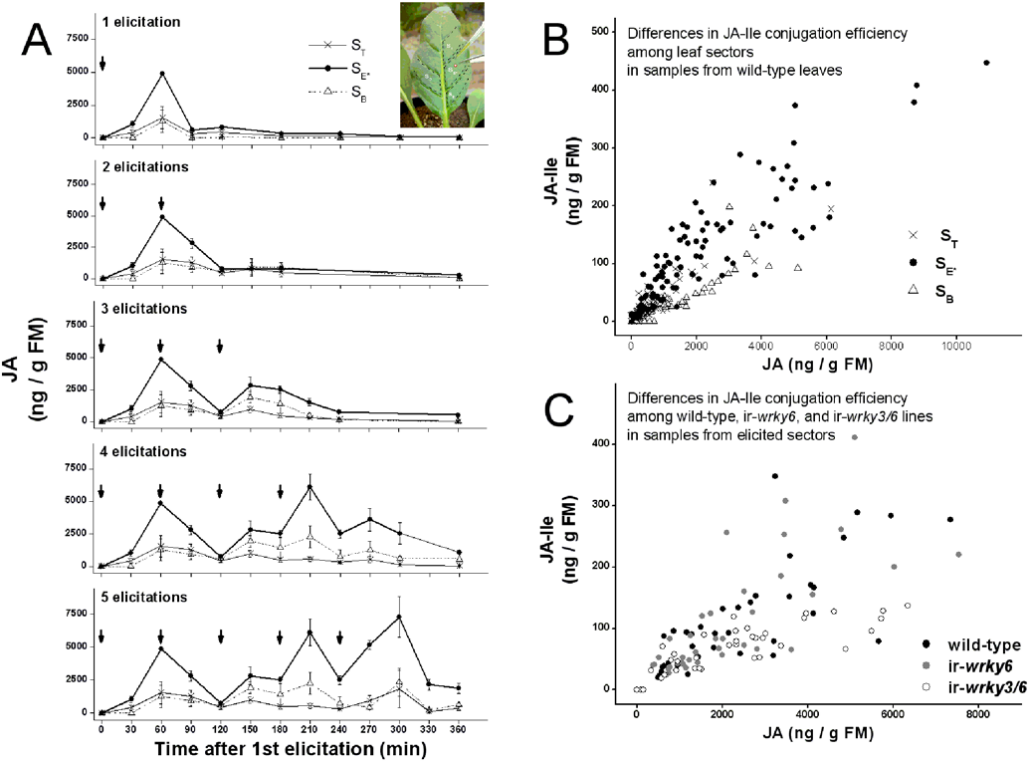
Spatial heterogeneity in JA accumulation and metabolism within an elicited leaf after repeated OS-elicitations. (A) JA accumulation in the repeatedly elicited laminal sector (S_E*_, closed circles on thick solid lines) was 3 times the amount in adjacent, non-elicited sectors (S_B_, open triangles, dot-dash lines; and S_T_, x's. thin solid lines). Sectors separated from the elicitation site by the mid-rib (S_O_) rarely accumulated any JA (data not shown). Peaks and troughs in the JA kinetic are less distinct in non-elicited sectors on the elicited side of the midrib. (B) Kinetics of JA-Ile elicitation closely track the JA kinetics after repeated elicitations, but the ratio of JA-Ile to JA from all samples from S_B_ [open triangles, m = 0.0286±0.00138 (S.E.)] was significantly lower than those in S_E*_ [closed circles; 0.0414±0.00199 (S.E.); Student's *t*-test, P≪0.0001] and S_T_ [x's; 0.0388±0.00217 (S.E.); Student's *t*-test, P = 0.0001]. (C) Silencing the expression of both *Na*WRKY3 and *Na*WRKY6 significantly reduced elicited JA-Ile to JA ratios in elicited laminal sectors [ir-*wrky3/6*; open circles; m = 0.0187 (S.E.)±0.00258] compared to wild-type plants [closed circles; 0.0381±0.00100 (S.E.); Student's *t*-test, P≪0.0001] and ir-*wrky6* plants [gray circles; 0.04285±0.00729 (S.E.); Student's *t*-test; P = 0.003]. Arrows indicate time of elicitation (1 per h).

Mean JA in both **S_B_** and **S_T_** 60 min after 1 elicitation was less than 1/3 of that in S_E*_ (Fig. 5A). Mean JA-Ile in those sectors at the same time point was less than 1/5 of S_E*_. The difference in JA between S_E*_ and the distally adjacent sectors briefly diminished after the 2^nd^ and 3^rd^ elicitations, but after the 4^th^ and 5^th^ elicitations the differences again increased so that peak JA means in **S_B_** and **S_T_** were again less than 1/3 of those in S_E*_. Separation of the analysis site from the elicitation site by the midrib inhibited the spread of the JA burst: only 2 of the 112 samples analyzed from **S_O_** contained detectable levels of any of the 5 jasmonates analyzed. In addition to being quantitatively lower than in S_E*_, JA and JA-Ile kinetics during the 5 elicitations in **S_B_** and **S_T_** were qualitatively less dynamic: peaks and troughs in the distal sector kinetics were not as distinct. 12/11-OH-JA, 12/11-OH-JA-Ile, and 12-COOH-JA-Ile were also detected in **S_B_** and **S_T_**, but mean values were low and associated with high variance, and were not very responsive to repeated elicitations (data not shown).

### JA-Ile conjugation during the JA burst is sector-specific and regulated by WRKY transcription factors

JA and JA-Ile kinetics within sectors were closely synchronized, however, the difference in JA-Ile between distal sectors and S_E*_ were consistently greater than the difference in JA. We therefore analyzed the ratio of JA-Ile to JA in each sample to determine if conjugation differed among sectors. We regressed JA-Ile content from each replicate against JA content from each sample (Fig. 5B) and used the slope of the regressions to describe the efficacy of JA-Ile conjugation for a given an amount of JA. The calculated slopes differed significantly between S_E*_ and **S_B_** (Student's *t*-test, *P*<0.001) but not S_E*_ and **S_T_** (Student's *t*-test, *P* = 0.38).

Contrary to our initial hypothesis, plants silenced in *Na*WRKY3 and *Na*WRKY6 expression were not limited in the ability to accumulate JA within S_E*_ after repeated localized elicitations (data not shown). However, from the same regression analysis used to quantify JA-Ile conjugation efficacy above, we discovered that elicited S_E*_ sectors of *Na*WRKY3/6 silenced plants had JA-Ile conjugation efficiencies as low as those measured in the **S_B_** sectors of WT plants (Fig. 5C).

### Spread of the JA burst among leaf sectors is directional, but inconsistent

We noticed considerably higher variance in mean JA levels in distal sectors compared with those of S_E*_. In total, 28 harvests of each laminal sector were analyzed to create the jasmonate kinetics presented in this study; of these, 19 sample means in sectors **S_B_** and **S_T_** were associated with standard errors greater than 25% of the mean (*n* = 4). In contrast, 6 of the means from S_E*_ had relative standard errors greater than 25%. The number of JA means with relative standard errors greater than 50%: 11 in **S_B_**, 8 in **S_T_**, and 1 in S_E*_ (means and variances from **S_O_** are not informative as only 2 of the 112 total replicates contained detectable levels of JA).

**Figure 6:**
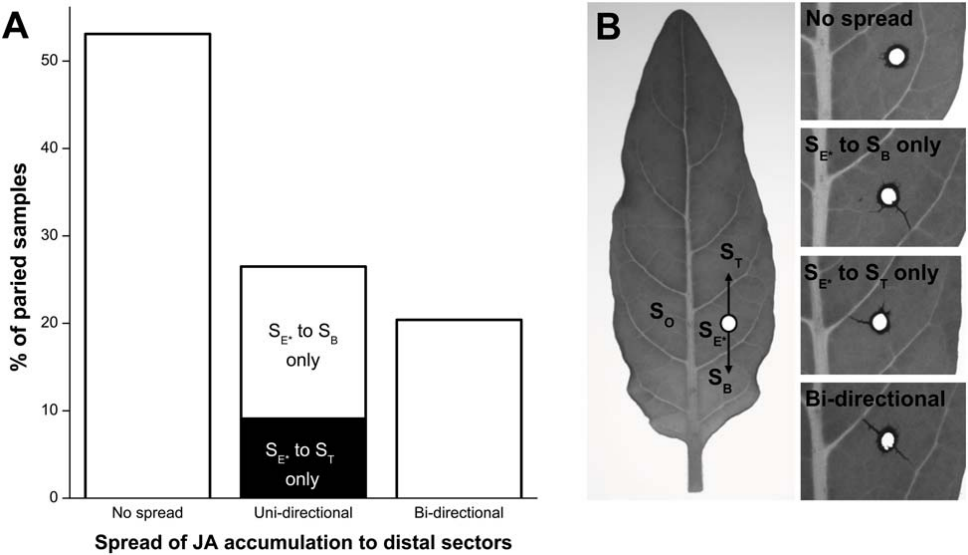
Spread of the JA burst from elicited to adjacent unelicited laminal sectors within a leaf were highly variable and likely depended on the connectivity of minor vasculature across sectors. (A) Of the 98 elicited leaves that accumulated JA in the elicited sectors (S_E*_) approximately half did not accumulate more than 500 ng/g JA in either of the adjacent, non-elicited sectors located proximal or distal to S_E*_ (S_B_ and S_T_), while the others accumulated JA in either S_B_, S_T_, or both adjacent sectors. Only 2 of the 112 leaves accumulated detectable quantities of JA in S_O_ (data not shown), the sector on the opposite side of the midrib from S_E*_. (B) Crystal violet dye (2%) applied to the elicitation sites allowed the visualization of apoplastic transport within leaves. Dye either was (1) not taken up, or transported (2) towards the base of the leaf, (3) the tip of the leaf, or (4) in both directions.

Reviewing the replicates contributing to each sample mean, we noticed a pattern that could be explained by vascular transmission of the JA burst: some replicates from distal sectors did not accumulate any detectable JA at all, while some replicates from the same sample group accumulated JA levels as high as those in replicates from S_E*_. Furthermore, some samples from **S_B_** accumulated JA levels similar to those in S_E*_, while samples from **S_T_** on the same leaf did not accumulate JA, and vice versa. Because every sample we analyzed was paired with samples from different sectors on the same leaf, we were able to look for directional patterns in the spread of the JA bursts within leaves (Fig. 6A). More than half of the time, JA did not accumulate substantially in either distal sector (less than 500 ng/g FM; 52 paired samples out of 98 total; 14 paired samples were not included because no jasmonates were detected in sector S_E*_ of the leaf in these samples). The remaining samples were divided between leaves in which JA accumulated above 500 ng/g FM in both **S_B_** and **S_T_** (“bi-directional”; 20 paired samples) and leaves where JA accumulated above that threshold in one but not in the other (“uni-directional”; 26 paired samples). To account for spurious differences due to an arbitrary “low” threshold of 500 ng/g FM, paired samples had to differ by at least a factor of 3 to be considered “uni-directional.” Additionally, 2 sample pairs were considered “uni-directional” because they differed by a factor of 4, even though JA in both samples was above 500 ng/g FM. Among those paired samples exhibiting uni-directional accumulation of JA, accumulation in **S_B_** occurred almost twice as often as in **S_T_**.

Because on this unanticipated level of inconsistency, we attempted to determine whether the connectivity of the minor vascular network spanning the laminal sections could play a role in the observed directional spread of JA accumulation. We applied a 2% crystal violet dye to needlepoint wounds like those used in our elicitation method (Fig. 6B). Needlepoint wounds that ruptured minor veins resulted in apoplastic uptake of the dye in the vein that was ruptured. Based on the location of the wound, apoplastic uptake of dye either (1) did not occur, or moved (2) toward the base of the leaf, (3) toward the tip of the leaf, or (4) in both directions (Fig. 6B). Dye was never observed moving across the midrib.

### OS-elicitation causes 1^st^ instar M. sexta to move and initiate feeding in unelicited sectors

**Figure 7:**
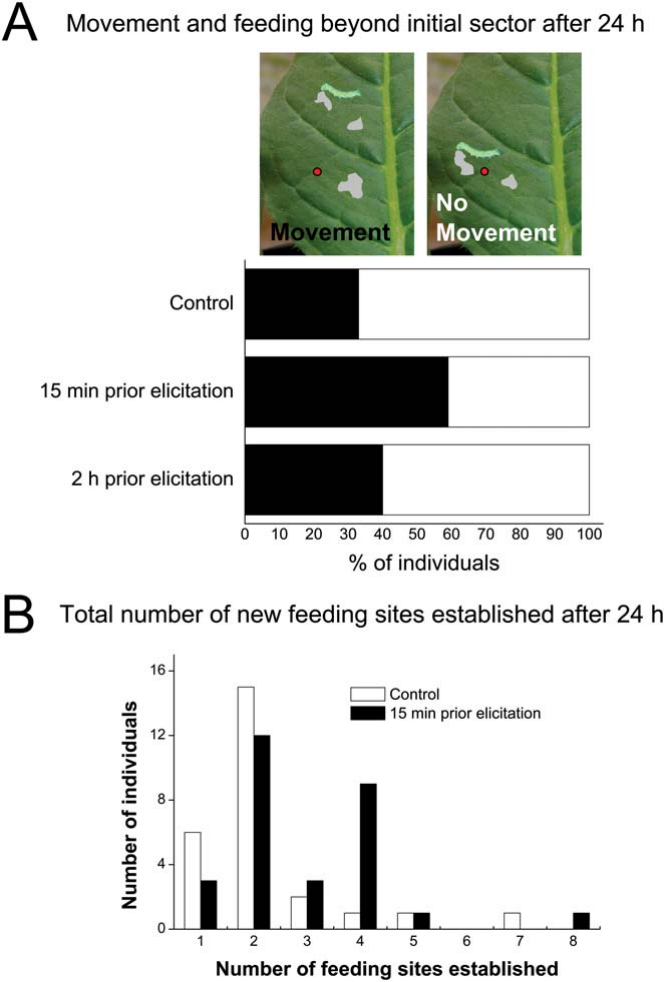
OS-elicitation motivates movement of *M. sexta* larvae away from elicited laminal sectors. 1^st^ instar *M. sexta* were placed on the underside of *N. attenuata* leaf sectors that were (1) un-elicited (control), or elicited (2) 15 min or (3) 2 h prior to placement of caterpillars. Movement and feeding activity of larvae between and within sectors were recorded. (A) 24 h after elicitation, a larger percentage of larvae had moved to a new laminal sector (solid bars) if the leaf had been elicited 15 min prior to placement (17 of 29 larvae) than if the leaf was un-elicited (9 of 27 larvae; pair-wise χ^2^ = 3.60, P = 0.06). However, if leaves were elicited 2 h prior, larval movement did not differ from that on control leaves (10 of 25 larvae moved to a new laminal sector). (B) Comparison of the total number of feeding sites established by individuals on control leaves (open bars) and leaves elicited 15 min prior to larval placement (solid bars) revealed that prior OS elicitation motivated larvae to initiate additional feeding sites on elicited leaves.

24 h after elicitation of focal sectors on *N. attenuata* leaves, a larger percentage of 1^st^ instar *M. sexta* larvae had moved away from leaf sectors elicited 15 min prior to initial feeding than from unelicited sectors (Fig. 7A; pair-wise χ^2^ = 3.60, P = 0.06). However, the number of individuals moving from sectors elicited 2 h prior to feeding was not significantly different than from unelicited sectors (pair-wise χ^2^ = 0.25, P = 0.62). The difference in individuals moving from sectors elicited 15 min and 2 h prior to feeding was not statistically distinguishable (pair-wise χ^2^ = 1.86, P = 0.17), but the potential gradient and overlap of these treatments at the molecular level makes this comparison difficult to decipher. Increased movement was associated with increased feeding initiation: more individuals on leaves elicited 15 min prior to feeding established more new feeding sites than those from other treatments (Fig. 7B).

### Repeated elicitations uncouple the association of JA/JA-Ile bursts with TPI activity

**Figure 8:**
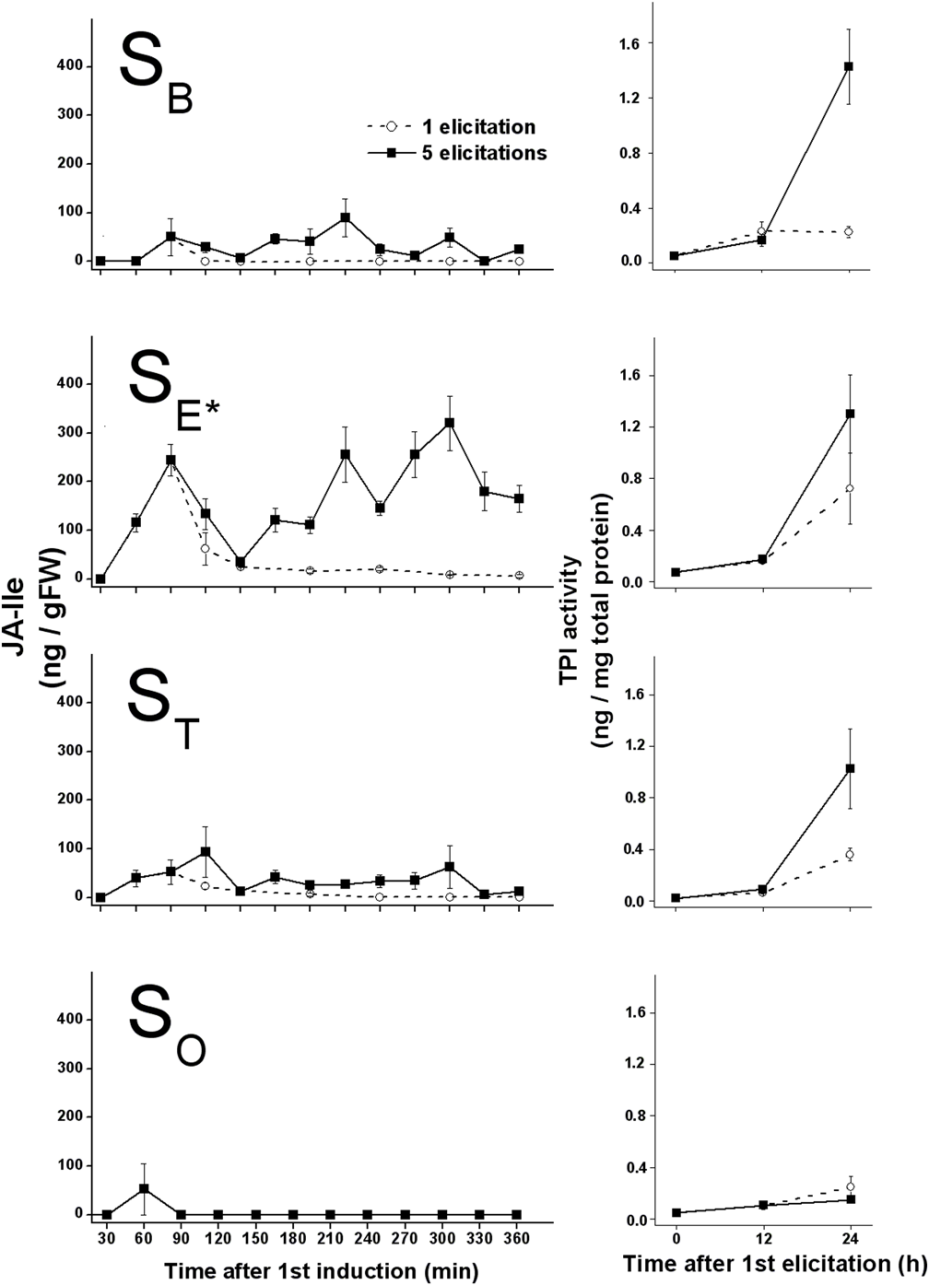
Early JA-Ile accumulations and later TPI activity in leaf sectors elicited once and five times reveals the proportionally of the responses. Increases in TPI activity (right column) in response to a single elicitation (open circles on dotted lines) are proportional to early increases in JA-Ile accumulation (left column): TPI activity differs among laminal sectors, with S_E*_ having significantly higher TPI activity than S_O_ and S_B_ (Fisher's PLSD, P<0.05) and marginally significantly higher TPI activity than S_T_ (Fisher's PLSD, P = 0.10). However, TPI activities after 5 successive elicitations (1 per h; solid squares on solid lines) do not differ significantly among S_B_, S_E*_, and S_T_ (ANOVA, F = 0.48, P = 0.63), although all values are higher than those elicited by a single elicitation in the respective laminal sectors. In S_O_, only 1 of the 68 analyzed sectors contained detectable JA-Ile and TPI activity did not differ from un-induced levels. Values represent JA-Ile or TPI activity mean±S.E. (n = 4 and 5, respectively).

Within-leaf TPI activity 24 h after a single elicitation tracked the pattern of JA-Ile accumulation among the different sectors, with S_E*_ having significantly higher TPI activity than **S_O_** and **S_B_** (Fig. 8; Fisher's PLSD, P<0.05) and marginally significantly higher TPI activity than **S_T_** (Fisher's PLSD, P = 0.10). However, repeated elicitations increased TPI activity in **S_B_** and **S_T_** to the same levels observed in S_E*_ after the 1^st^ elicitation, resulting in homogeneously high TPI activity, but only on the elicited half of the leaf (ANOVA of 24 h TPI activity in **S_B_**, S_E*_ and **S_T_** after repeated elicitations; F = 0.48; P = 0.63). TPI activity remained at un-induced levels in **S_O_**, demonstrating that the midrib blocked the transmission of the TPI-eliciting signal.

## Discussion

We characterized the spatial and temporal dynamics of the JA and JA-metabolite bursts in response to single and repeated OS-elicitations. We found that the metabolism and integration of the JA burst depends strongly on both the number of elicitations and the spatial location of the elicitation within a leaf, as well as *NaWRKY3* and *6*, factors which may function in the formation of plant memories that tailor the JA-mediated defense responses in the face of repeated challenges. Three aspects of this work, namely the temporal and spatial heterogeneity of the elicited bursts and their functional consequences, deserve additional discussion.

### Temporal integration of repeated elicitations as evidence of memory formation

Memory formation of past herbivory can prime plants for a more effective induced response to subsequent attack [49]–[52]. In particular, repeated elicitations from a continuously feeding herbivore could provide information about the likelihood of herbivory in the immediate future. Memory formation could occur when the discrete JA bursts elicited by repeated herbivory modify: (1) the time to attain maximum JA accumulation; (2) JA amplitude; or (3) baseline of accumulated JA [53].

Each of several repeated elicitations did indeed result in more responsive JA accumulations in S_E*_ than predicted from the additive effects of individual, “naïve” elicitations. The observed immediate increase in net JA after each elicitation (or the less-than-predicted decrease after the 2^nd^ elicitation) can be attributed to faster-than-predicted JA biosynthesis and/or slower-than-predicted metabolism. The polar JA metabolite 12-OH-JA rapidly accumulates in the vasculature in *A. thaliana* [16]. The net 12/11-OH-JA rates observed here could be explained by a dynamic between hydroxylation of JA and vascular loading. The immediate increases of 12/11-OH-JA after the 2^nd^, 3^rd^, and 4^th^ elicitations suggest that this route of JA metabolism is not negatively regulated by successive elicitations. If 12/11-OH-JA patterns are indicative of continued or increased JA metabolism after successive elicitations, then increases in net JA (and less-than-predicted decreases) must result from immediate, rapid biosynthesis of JA after each successive elicitation. Post-translational modifications of proteins (or the lack thereof), including continued activity of biosynthetic enzymes from the previous elicitations or deactivation (but not degradation) of such enzymes, are possible mechanisms of memory formation that could allow plants to increase the speed of defense deployment after successive elicitations [54], [55].

However, the most dramatic evidence of a memory effect was seen in the amounts of JA accumulated in response to 2 elicitations. Unlike any other elicitation, the 2^nd^ elicitation resulted in the complete absence of a discernable JA burst in both the whole-leaf and localized elicitations. The absence of a JA burst could reflect a limitation or repression of JA biosynthesis due perhaps to the timing of the 2^nd^ elicitation—which occurred during the peak accumulation of JA from the 1^st^ elicitation. Additionally, the 2^nd^ elicitation could elicit metabolism of JA that outpaces biosynthesis, by expressing or activating enzymes responsible for hydroxylation or conjugation to amino acids. As noted above, all elicitations induced an increase in 12/11-OH-JA accumulation, but it was unclear how much of synthesized JA this metabolite could account for. Like JA, JA-Ile decreased after a 2^nd^ elicitation, and accumulation of 12/11-OH-JA-Ile and 12-COOH-JA-Ile was not significantly increased by a 2^nd^ elicitation (Fig. 4B and 4C). However, the contribution of individual metabolites to changes in JA accumulation is difficult to determine due to the potential for further metabolism and vascular loading, processes that could also be subject to reconfiguration by repeated elicitations. Because of these complex and simultaneous interactions, the exact timing of repeated elicitations could play a critical role in determining whether JA accumulation increases or decreases.

Repeated elicitations increased baseline JA levels in S_E*_, but the increase was not directly proportional to the number of elicitations (Fig. 3B). Instead, JA (and JA-Ile) basal levels increased in discrete steps. JA returned to the same baseline 2 h after 1, 2, and 3 elicitations (∼1000 ng/g FM), and then returned to a higher baseline after 4 and 5 elicitations (∼2500 ng/g FM). The JA-Ile baseline increased substantially after the 2^nd^ and 4^th^ elicitations. These elevated baseline levels were not permanent: for example, increased JA and JA-Ile baselines held constant for about an hour after the burst induced by 4 elicitations, then declined rapidly to lower levels. However, neither the stepwise elevation nor the eventual decline was accounted for simply by changes in hydroxylation; 12/11-OH-JA increased after the 4^th^ elicitation and then slowly declined, as did 12/11-OH-JA-Ile after the 2^nd^ and 4^th^ elicitations.

These results demonstrate that the JA burst does not simply accumulate in response to repeated elicitations as the sum of single elicitation responses and that evidence for all three expectations for the signatures of memory formation was found. The number of metabolic outlets for JA makes it difficult to determine a mechanism for the accumulation patterns reported here; however, the accumulation patterns of several different jasmonates in response to repeated elicitations suggests that memory formation can reconfigure the JA signaling network at multiple nodes. Because of the regulatory function of the jasmonates, the specific patterns of JA accumulation due to this memory formation has the potential to further tailor the plant defense response to continuous herbivory.

The choice of elicitation frequency used in this experiment (1 per h) was motivated by the specific characteristics of *N. attenuata*'s JA burst so that subsequent elicitations would occur when the JA burst in response to a single elicitation had attained maximum values. The elicitation frequency from the feeding behavior of *M. sexta* larvae will change as larvae develop through their 5 instars [56]. The integration of the elicitations will likely change with the changing kinetics of feeding bouts. If the patterns of JA and JA-Ile accumulation described here differ with changes in the frequency of elicitation, it is possible that the elicited defense response could be tailored not only to the species of herbivore, but the developmental stage of a specific herbivore. *M. sexta* larvae cause the most damage to *N. attenuata* plants during the 5^th^ instar, when they consume >98% of their total leaf tissue intake; by comparison, early-instar caterpillars are relatively benign, causing only minor amounts of damage. Furthermore, larvae between the second and third instars have usually attained sufficient mass to move among plants [38]. If the induced defenses of plants are sufficiently powerful to motivate larvae to switch plants, and plants are able to recognize the particular feeding frequency of 3^rd^ instar larvae, they might be able to turn their herbivore pests into offensive weapons that reduce the competitive ability of their neighboring conspecifics [27], [38].

### Vascular architecture constrains and organizes the spread of the JA burst and its maturation into a JA-Ile burst

Given that vascular connectivity is known to influence the elicitation and distribution of systemic defenses [18] and that JA biosynthetic enzymes are specifically localized in the vascular bundles in tomato leaves [57], we were surprised to find that *N. attenuata*'s vasculature constrained, rather than propagated, the JA bursts from a point source elicitation within an elicited leaf. This constraint was not seen in a previous study that examined whether *M. sexta* larvae could consume the JA burst that its feeding activity generated, simply because in that study the OS elicitation treatment was applied to the edge of an incision that ran parallel to the midrib and thereby elicited all laminar sectors on the treated side of the midrib [39]. Realizing that secondary veins constrain the spread of the JA burst into the adjacent sectors, we now must change the conclusions reached by Schittko et al. and recognize that it is indeed possible for a voracious larvae to consume the JA burst that its feeding generates within a sector, and to easily move to an un-elicited adjacent sector on the opposite side of the midrib.

The statistical analysis of JA bursts in adjacent sectors on the same side of the midrib revealed a pattern of between-sector spread, which is likely explained by transmission of a damage signal through minor vascular elements that connect these adjacent sectors. Avoiding damage to these minor vascular elements could be an effective strategy for an herbivore to employ in order to limit the extent of JA accumulation and prevent it from outpacing its own feeding.

The high variability and inconsistent directionality of JA accumulation in **S_B_** and **S_T_** suggest that, although the secondary vasculature is not an absolute barrier to the spread of JA accumulation within a leaf, the spread of the purported signal responsible for initiating JA biosynthesis in sectors distal to the elicitation site requires a path to travel through the differentiated vascular tissue. Vascular apoplastic uptake of dye applied to needlepoint wounds showed that the connectivity of the wound site to minor vasculature—specifically, the physical rupturing of a minor vein—determines the subsequent directionality of applied fluids; this connectivity of minor vasculature may be a mechanism determining directionality of JA accumulation. Additionally, *M. sexta* OS are known to contain fatty acid-amino acid conjugates (FACs) that elicit specific responses to herbivory in *N. attenuata*, even when diluted 1∶1000 [39]. Apoplastic transport of FACs could further complement the directionality of JA accumulation.

It is particularly interesting that different sectors conjugate the JA burst into JA-Ile differentially. This differential response suggests that, in addition to the spatial heterogeneity resulting from vascular constraints, different regulatory pressures are mediating the plant defense response in different parts of the leaf. We found that simultaneously silencing *Na*WRKY3 and *Na*WRKY6 expression limits JA-Ile conjugation after elicitation in a similar manner as the basal leaf sector distally adjacent to an elicited leaf sector in wild type leaves. It is unclear if the differential spatial response is dependent on the type of tissue, or if moving the elicitation site would change the spatial differences in conjugation efficiency. However, if JA-Ile is indeed the active elicitor of JA-induced defense responses, then this difference should have profound functional consequences for how different sectors defend themselves.

### Functional significance of the JA bursts and their spatial heterogeneity

Here, we report the first evidence that *M. sexta* larvae are repelled by rapidly elicited responses that correlate with the occurrence of JA bursts: larvae moved and established new feeding sites in un-elicited laminal sectors. This behavior was not as prevalent in *M. sexta* that fed on leaves elicited 2 h prior to the experiment, suggesting that the caterpillars could be responding to a cue specifically associated with the early plant defense response; this timeframe coincides with peak accumulation of JA and JA-Ile. The differences in movement among treatments were detectable after 24 h, at which time *M. sexta*-induced TPI activity in *N. attenuata* is detectable, but has not yet reached its peak [58]. Previously, a direct role for JA in mediating plant-herbivore interactions, independent of other JA-dependent defenses was identified: *Helicoverpa zea* larvae activated cytochrome P450 genes associated with detoxification of allelochemicals when fed an artificial diet containing large amounts of MeJA [59]. The robustness of the JA burst in a native population of *N. attenuata* reported here underscores the fact that *M. sexta* will consistently encounter JA bursts when feeding on these plants, and suggests that the JA burst itself could function defensively. Substantial additional work will be required to determine if the larvae are responding specifically to the JA burst or some other rapidly elicited response that co-occur with the JA bursts.

Adler and Karban posit that variability in the plant defense response can be more effective than optimally induced or constitutive defenses under certain conditions (the Moving Target Model of defense) [32]. The Moving Target Model proposes that induced plant defense phenotypes cannot be set on a single axis from least costly, least effective to most costly, most effective. Rather, acknowledging the multiple components contributing to a change in plant phenotype and the range of effects a given defense might have on a particular herbivore, the model suggests that the induced plant defense response may appear random from the herbivore's perspective. This perceived randomness may have a defensive function in itself, and the model predicts that resistance to herbivory is correlated with the amount of phenotypic variability of defense rather than an axis of defense states and corresponding costs. The heterogeneity of JA accumulation within elicited leaves that we report here is an example of an “unpredictable” plant defense response to herbivory; each elicited leaf has a different patchwork pattern of JA accumulation based on certain constraints (such as vascular connectivity and conjugation efficiency). Understanding these constraints could lead to prediction of the pattern of JA accumulation, but the response may appear unpredictable to a feeding herbivore. Because *M. sexta* move away from recently elicited tissue, the effectiveness of such heterogeneity in this case would be determined by how well the herbivore chooses from the remaining, “randomly” induced or un-induced tissue.

Here, TPI activity 24 h after elicitation appeared to be subject to similar spatial constraints affecting JA accumulation after a single elicitation (Fig. 8). However, repeated elicitations within the same laminal sector overcame the spatial constraints so that mean TPI activity was similar in all sectors on the same side of the mid-rib; this result contrasts with JA/JA-Ile accumulation patterns. TPI activity in *N. attenuata* is known to be strongly influenced by the *Na*JAR4-mediated conjugation of Ile to JA [25], [30], and the decoupling of JA-Ile accumulation and TPI activity after repeated elicitations was unexpected. Clearly, some amount of JA/JA-Ile accumulation is necessary for later TPI activity: the non-induction of TPI activity in **S_O_** (separated from the elicitation site by the midrib) reflects the near-complete absence of any accumulation of jasmonates, even after repeated elicitations. JA-Ile could be a particularly potent signal for JA-responsive gene expression, and TPI activity levels may be responsive to trace amounts of the molecule. JA-Ile-dependant JAZ degradation was unaffected in the *A. thaliana jar1-1* mutant accumulating 25% of wild-type JA-Ile levels [28]; this JA-Ile “leaking” effect was echoed by an independent study [60]. In that case, what might be more important for induction of TPI activity is the duration of a minimum JA-Ile dose achieved in both local and distal sectors by repeated elicitations, rather than the amount. One notices, for example, that JA-Ile levels in S_E*_ remains above 20 ng/g FM for almost 5 h after a single elicitation; TPI activity levels in that sector are more similar to those after 5 elicitations. For this hypothesis to account for the spatial differences in TPI activity after a single elicitation we see here, and the lack of difference after 5 elicitations, the effective potency of JA-Ile would have to be near 20 ng/g FM (or ∼300 ng/g FM for JA). As suggested by Chung et al., an alternative explanation is the existence of a supplementary bioactive molecule, perhaps JA or another member of the jasmonate family [28].

The jasmonate signaling network mediating the induced defense response interacts with other phytohormones at several nodes to tune the defense response [61], and whether JA-Ile functions at the suggested potency level *in vivo* or not, induction of TPI activity within a leaf could be subject to other modifications or crosstalk that contribute to the patterns reported here. For instance, TPI expression in tomato is regulated by abscisic acid and ethylene in addition to JA [62], [63]. Furthermore, TPI activity is several steps removed from the transcription of TPI mRNA and is therefore subject post-transcriptional and post-translational modification. To date, no studies have investigated how such processes might vary in sites spatially distal to an elicitation site within an elicited leaf. Given the resolution of heterogeneity that we report in JA accumulation, this could be a fruitful line of research to follow.

Finally, TPI activity is a robust marker of the herbivore-induced, JA-dependent defense response, particularly in the context of Lepidopteran feeding [64], but cellular accumulation of JA is associated with many changes in the composition of herbivore-attacked tissue. These changes are not limited to induction of so-called secondary metabolites; herbivore and pathogen attack result in a reconfiguration of primary metabolism as well [65]. Other JA-related responses may follow more closely the spatial heterogeneity and temporal build-up of jasmonates reported here, even after repeated elicitations. Parsing out the temporal dynamics and spatial heterogeneity of the induced defense response within a leaf will shed light on induced plant defenses that occur during critical windows of Lepidopteran herbivory, but elicitation methods must be targeted to the appropriate spatial and temporal scale.
